# Ganglioside Composition Distinguishes Anaplastic Ganglioglioma Tumor Tissue from Peritumoral Brain Tissue: Complementary Mass Spectrometry and Thin-Layer Chromatography Evidence

**DOI:** 10.3390/ijms22168844

**Published:** 2021-08-17

**Authors:** Dragana Fabris, Ivana Karmelić, Hasan Muharemović, Tomislav Sajko, Mia Jurilj, Slavica Potočki, Ruđer Novak, Željka Vukelić

**Affiliations:** 1 Department of Medical Chemistry, Biochemistry and Clinical Chemistry, School of Medicine, University of Zagreb, Šalata 3, 10000 Zagreb, Croatia; ivana.karmelic@mef.hr (I.K.); slavica.potocki@mef.hr (S.P.); 2 Department of Physical Chemistry, Ruđer Bošković Institute, Bijenička cesta 54, 10000 Zagreb, Croatia; hmuharem@irb.hr; 3 Department of Neurosurgery, University Hospital Center “Sestre Milosrdnice”, Vinogradska cesta 29, 10000 Zagreb, Croatia; neurosajko@gmail.com (T.S.); mia.jurilj@gmail.com (M.J.); 4 Department for Protemics, Center for Translational and Clinical Research, School of Medicine, University of Zagreb, Šalata 11, 10000 Zagreb, Croatia; rudjer.novak@mef.hr

**Keywords:** ceramides, ganglioglioma, gangliosides, mass spectrometry, sphingolipids

## Abstract

Gangliosides serve as antitumor therapy targets and aberrations in their composition strongly correlate with tumor growth and invasiveness. Anaplastic ganglioglioma is a rare, poorly characterized, malignant neuronal–glial tumor type. We present the first comparative characterization of ganglioside composition in anaplastic ganglioglioma vs. peritumoral and healthy brain tissues by combining mass spectrometry and thin-layer chromatography. Anaplastic ganglioglioma ganglioside composition was highly distinguishable from both peritumoral and healthy tissue despite having five to six times lower total content. Ten out of twelve MS-identified ganglioside classes, defined by unique glycan residues, were represented by a large number and considerable abundance of individual species with different fatty acid residues (C16–C24) in ceramide portions. The major structurally identified class was tumor-associated GD3 (>50%) with 11 species; GD3 (d18:1/24:0) being the most abundant. The dominant sphingoid base residue in ganglioside ceramides was sphingosine (d18:1), followed by eicosasphingosine (d20:1). The peritumoral tissue ganglioside composition was estimated as normal. Specific ganglioside composition and large variability of ganglioside ceramide structures determined in anaplastic ganglioglioma demonstrate realistic ganglioside expression patterns and correspond to the profile of high-grade malignancy brain tumors.

## 1. Introduction

Ganglioglioma are benign (WHO grade I) [1] and rare well-differentiated and typically slow-growing mixed glioneuronal tumors composed of dysplastic mature ganglion cells and neoplastic glial cells with the highest incidence rates being in children and young adults, but they can occur at any age and anywhere in the central nervous system [2,3,4]. They are mainly characterized by a space-occupying lesion in the temporal and frontal lobes and are usually associated with chronic drug-resistant epilepsy [3,4,5]. The standard treatment is complete surgical resection [4,6], but even then ganglioglioma can develop a malignant transformation into anaplastic ganglioglioma (AGGL) classified as a grade III tumor [1,7]. AGGL is a rare tumor only occasionally occurring de novo. Its incidence is only 0.02 cases/million/year [8,9]. It is poorly characterized, with the literature limited to case reports and small series. AGGLs are composed of dysplastic ganglion cells and an anaplastic glial component with increased mitotic activity, pleomorphism, microvascular proliferation, necrosis, and a gemistocytes differentiation pattern [3,10]. AGGL is highly epileptogenic and associated with strong potential for distant relapse and short overall survival [11].

Lipidomic remodeling and dysregulation of lipid metabolism are recognized as hallmarks of malignant transformation in many different cancer cells [12]. Gangliosides (GGs) are sialylated glycosphingolipids (GSLs) that present as integral structural components of the cell membrane, including microdomains—lipid rafts, where they function as cell-surface markers and antigens, but also as molecules regulating various signals [13]. GGs are composed of a hydrophobic ceramide backbone embedded in the outer layer of the cell membrane while the hydrophilic carbohydrate residue participates in the formation of glycocalyx on the cell surface [14]. Their bioactive metabolites ceramide (Cer) and long-chain sphingoid bases, particularly sphingosine (d18:1) and sphingosine-1-phosphate (S1P), regulate cell signaling as second messengers and control numerous processes such as cell growth, migration, differentiation, and apoptosis [15]. There is more and more evidence showing that different ceramide species have distinct cellular functions; C16- and C18-ceramides (containing sphingosine linked by amide bond to palmitic acid, C16:0, or stearic acid, C18:0) are associated with induction of apoptosis, while ceramides with longer fatty acid chains (C22-, C24-) show antiapoptotic properties [16,17]. The mechanism by which they influence cell behavior has not been elucidated yet, but probably involves interfering with signaling pathways [18].

Brain tumors are characterized by irregular GG metabolism and changes in GG expression strongly affect cellular growth, adhesion, and invasiveness of tumor cells [19,20]. These modifications of cell-surface glycosylation lead to the expression of cell-surface antigenic structures that are strongly associated with metastatic potential and invasiveness of tumor cells [21]. Antitumor-associated GG monoclonal antibodies have been developed for diagnosis, monitoring and treatment of cancer patients [22].

According to our knowledge, no reports on the (glyco)sphingolipid composition of anaplastic ganglioglioma have been published so far. Since AGGL represents a very rare type of mixed glioneuronal tumor, we believe that applying a similar approach in glycosphingolipidomic mapping of this tumor as we used in our previous analysis could contribute to our understanding of GG roles in AGGL and also in brain tumors in general. In addition, we considered that the peritumoral tissue (PT) ganglioside content analyzed in this research could provide very valuable information since the biochemical changes that occur in the closest surroundings of the tumor are considered crucial for tumor cell infiltration into the surrounding healthy brain tissue and for promoting tumor recurrence [23]. However, the analysis of peritumoral brain areas has been highly neglected with only few performed so far with glioblastoma multiforme (GBM) peritumoral tissues [24]. The use of modern mass spectrometric techniques for detailed structural analysis of gangliosides in tumor and peritumoral tissues elucidates the realistic state of GG expression at the cell surface. 

## 2. Results

### 2.1. Quantitative Ganglioside Analysis

Complex ganglioside mixtures were isolated and purified from tissue samples in parallel. AGGL tumor tissue mass was 1.10 g, peritumoral brain tissue 0.97 g, and normal brain (NB) tissue 0.22 g. Total GG content was spectrophotometrically determined to be 96.4 μg of GG-bound sialic acids (SAs) per gram tissue wet weight (μg GG-SA/g tissue w.w.) in AGGL sample tissue, 447.0 μg GG-SA/g tissue w.w. in PT, and 545.2 μg GG-GA/g tissue w.w. in NB tissue. Total GG content in AGGL was approximately six times lower than in NB, while approximately five times lower than in the PT tissue.

### 2.2. High-Performance Thin-Layer Chromatography (HPTLC) Analysis of GG Mixtures 

**Figure 1:**
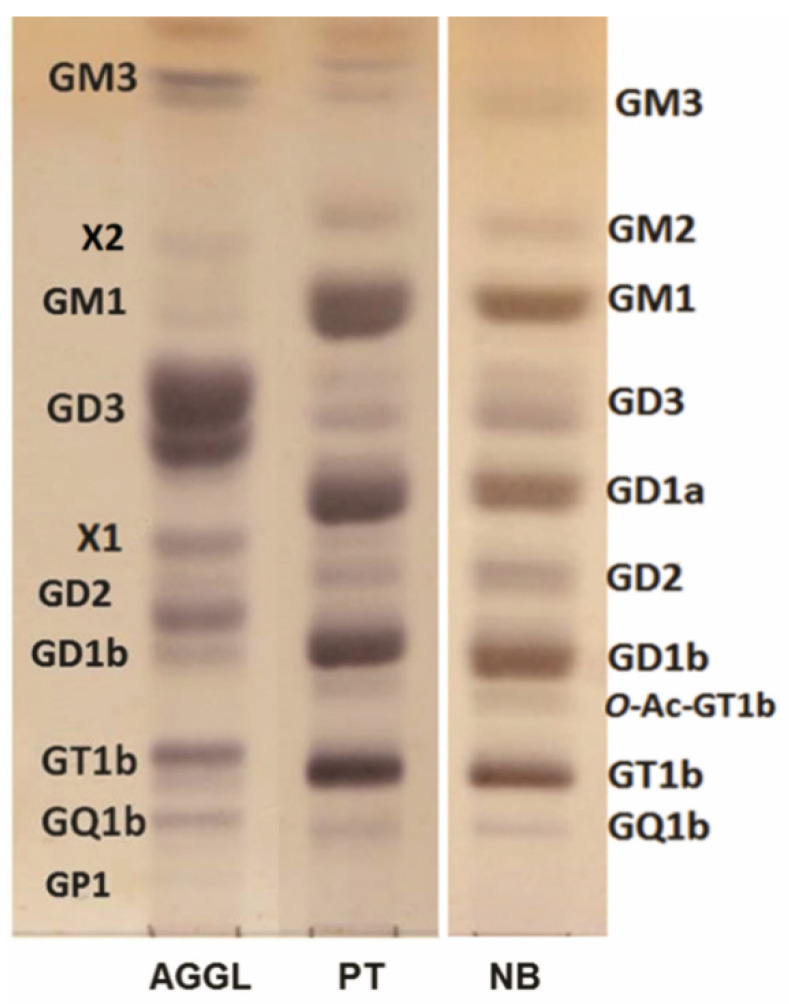
HPTLC-separated ganglioside fractions isolated from anaplastic ganglioglioma (AGGL), peritumoral tissue (PT), and normal brain tissue (NB), followed by resorcinol staining. X2—migrates as 3′-isoLM1; X1—migrates as GD1α; GM3 and GD3 fractions appear as double bands.

HPTLC was used for qualitative analysis and determination of relative proportions of AGGL, PT, and NB ganglioside fractions (Table 1, Figure 1). AGGL GG migrating fractions corresponding to GM3, GD3, GM1, X1 (possibly corresponding to GD1α), GD2, GD1b, GT1b, GQ1b, GP1, and X2 possibly corresponding to 3′-isoLM1, with some GG fractions specifically appearing as double bands (GM3, GD3, and GD2). The GM1-migrating fraction in AGGL corresponds to the lower part of the GM1-migrating fraction in PT. GD1a and *O*-Ac-GT1b migrating fractions were also detected in PT and NB tissue, but no X1, X2, or GP1-migrating fractions were detected. The GG pattern of HPTLC-separated fractions is considerably different between AGGL and PT tissues, with very high similarity present between PT tissue and NB tissue GG patterns (Table 1, Figure 1). The most abundant GG fractions in AGGL correspond to GD3 (upper and lower fractions), representing 52% of the total GG content (Table 1). Besides GD3, other GG fractions corresponding to “simple” GG structures such as GM3 and GD2 and, from more complex ones, X1 (possibly GD1α) were detected in significantly higher relative proportions in AGGL than in the PT and NB tissues (Table 1). More complex GG structures such as GM1, GD1a, GD1b, and GT1b were detected as the most abundant GG structures in PT and NB (as expected for adult human brains), while their abundance was considerably lower in AGGL tissue.

### 2.3. Compositional Analysis of AGGL, PT, and NB Tissue GG Mixtures via MS 

**Figure 2:**
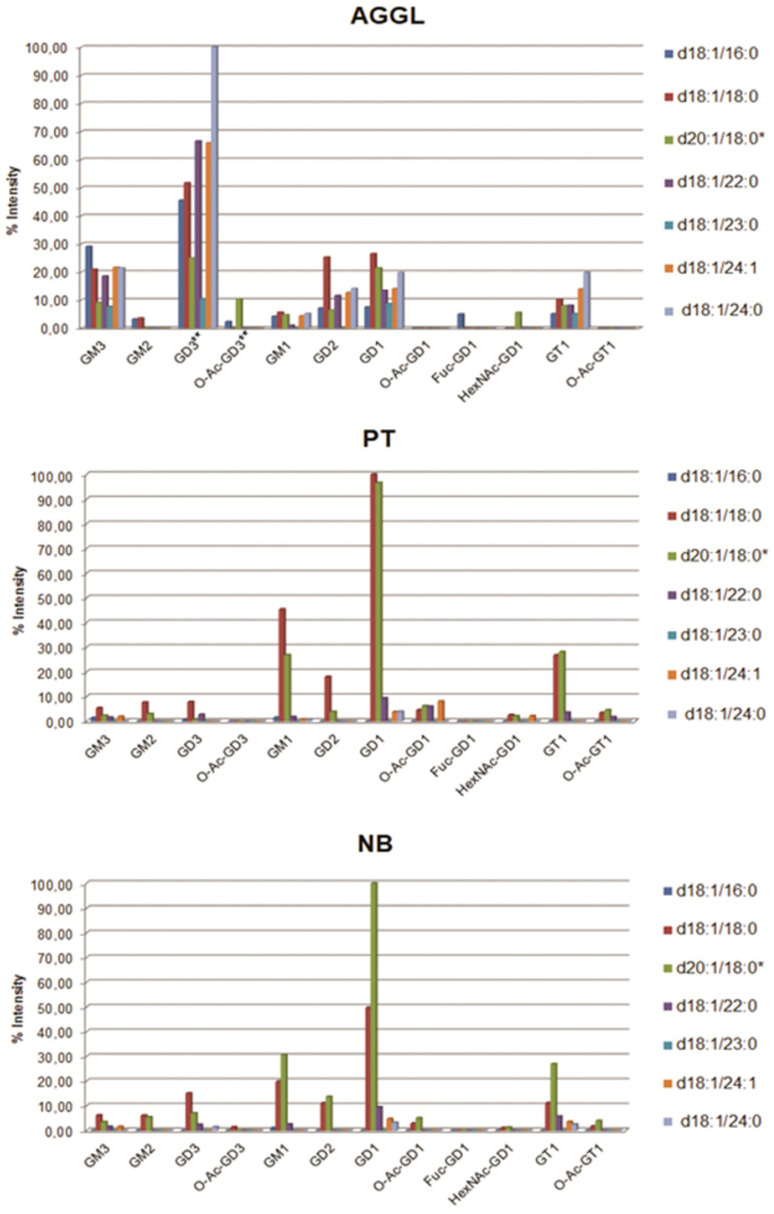
Ganglioside species characterized by variable ceramide structures and their relative abundances (relative ion intensities) detected via MS analysis of purified ganglioside mixtures from anaplastic ganglioglioma, peritumoral tissue and normal adult human brain tissue. * d20:1/18:0 and/or d18:1/20:0, ** *O*-Ac-GD3 (d20:1/18:0) and GD3 (18:1/23:0) in AGGL are present as a mixture; their relative proportions are roughly divided in half.

Native GG mixtures from AGGL, PT, and NB tissue samples were analyzed in parallel via MS in negative ion mode. The acquired mass spectra are shown in Figure S1 and corresponding ion lists in Tables S1–S3 (Supplementary Materials). The MS1 spectra verified the presence of all GG fractions detected via HPTLC, additionally revealing their variability in the ceramide part of the molecule. Figure 2 represents the compositions of GGs from AGGL, PT, and NB samples, including relative abundances of individual GG species defined according to the structural variability of their ceramide portion, as evidenced by combining MS and tandem MS analysis data. Intensities of molecular ions of GG species detected by MS1 are in good accordance with densitometrically determined relative abundances of GG fractions separated via HPTLC. If in MS1 screening (Tables S1–S3, Supplementary Materials) one ionized GG species appeared as two or three molecular ion forms (single-, double-, and/or triple-charged ion), the sum of individual ion intensities is given as total ion intensity of that GG species.

In the AGGL GG mixture, GD3 gangliosides with variable ceramide compositions were detected as the most abundant ions. The ion of highest intensity corresponded to GD3 with a d18:1/24:0 ceramide structure, followed by GD3 (d18:1/22:0), and GD3 (d18:1/24:1), as shown in Figure 2 and Tables S1 and S2 (Supplementary Materials). Other “simple” GG species also revealed by highly abundant ions were GM3 and GD2 with variable ceramide structures, while complex GG species GM1, GD1, and GT1 appeared as ions of much lower intensities. The ions corresponding to GD1 structures are probably representing the mixture of GD1b and GD1α (noted as X1 in HPTLC) since these isomers cannot be distinguished via MS.

All identified GG classes defined by unique glycan residues were represented by a large number of individual species with different ceramide portions. The ceramide portions diversity was primarily characterized by a larger number and a considerable relative abundance of different fatty acid residues: long-chain C16, very long-chain C22, C24, and C24:1, as well as long-chain C17 and C19 with an odd number of C-atoms. Ions corresponding to fucosylated GD1 with a d18:1/16:0 ceramide structure (Fuc-GD1) were detected exclusively in the AGGL GG mixture, while ions corresponding to HexNAc-GD1 structures were detected in higher concentration in AGGL than in PT and NB tissues (Figure 2, Tables S1–S3, Supplementary Materials).

In PT and NB tissues, the highest intensity ions corresponded to complex gangliosides GM1, GD1, and GT1 (as expected for adult human brain tissue) with the composition of ceramide structures limited to only two of the most common types, d18:1/18:0 and d20:1/18:0, composed of stearic acid residue (18:0) and two types of sphingoid bases, sphingosine (d18:1) and eicosasphingosine (d20:1), but the ions corresponding to GGs containing d20:1/18:0 ceramides most probably represented a mixture of GGs containing both d20:1/18:0 and d18:1/20:0 ceramide structures. Ions corresponding to simple GGs (GD3, GM3, and GM2) were also detected in PT and NB but with significantly lower intensities than in AGGL tissue. In PT tissue, GD1 and GT1 gangliosides shared similar contents of d18:1/18:0 and d20:1/18:0 ceramides, while in GM1 there was a higher content of d18:1/18:0 ceramide (Figure 2). In NB tissue, complex GGs GD1, GT1, and GM1 contained d20:1/18:0 ceramide as the most abundant; its relative abundance was up to 1.5 times higher compared to d18:1/18:0 in the corresponding GG species (Figure 2). On the contrary, in simple GGs GM3 and GD3, d18:1/18:0 ceramide residue was more abundant than that of d20:1/18:0. Another hallmark of both PT and NB tissues is the presence of *O*-Ac-GD1b and *O*-Ac-GT1b species (Figure 2; Tables S2 and S3 Supplementary Materials), known as “neurostatins” [25].

### 2.4. Structural Identification of AGGL Ganglioside Species via MS Sequencing

**Figure 3:**
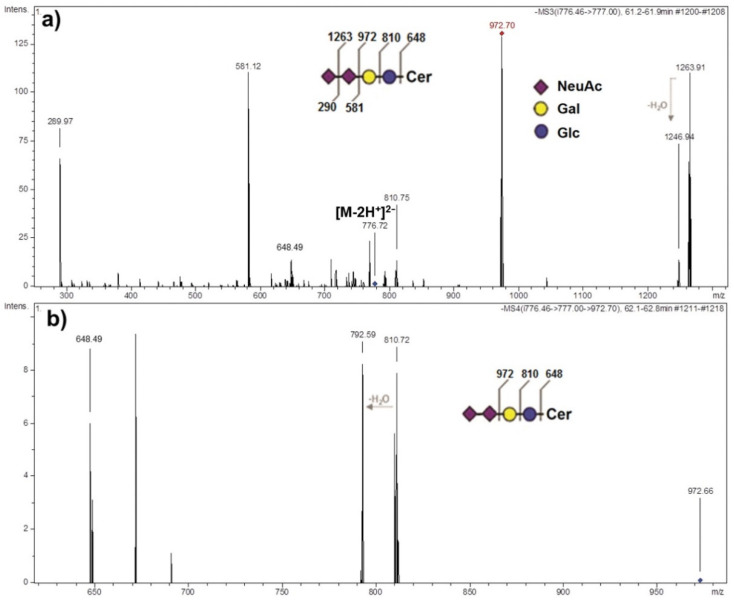
Fragmentation spectra obtained via multistage CID MS^2^ (**a**) and MS^3^ (**b**) of the [M-2H^+^]^2−^ ion at *m/z* 776.46 confirming the GD3 (d18:1/24:0) structure in the ganglioside mixture from anaplastic ganglioglioma tumor tissue.

The presence of ganglioside species GD3 with a d18:1/24:0 ceramide composition in the AGGL GG mixture was structurally confirmed via CID multistage sequencing (MS^2^ and MS^3^) analysis (Figure 3). The corresponding doubly charged molecular ion [M-2H^+^]^2−^ at *m/z* 776.72 detected by MS1 screening of the GG mixture was selected for MS^2^ sequencing. The product ion spectrum revealed the required diagnostic fragment ions: the monosialo fragment, NeuAc^−^ at *m/z* 289.97; the disialo fragment, NeuAc-NeuAc^−^, at *m/z* 581.12; the ion at *m/z* 1263.91 corresponding to the NeuAc-Gal-Glc-Cer^−^ sequence, produced by the loss of terminal sialic acid residue from the parent ion; the ion at *m/z* 972.70 corresponding to Gal-Glc-Cer^−^, the desialylated sequence (Figure 3a). The later ion at *m/z* 972.70 selected for further MS^3^ fragmentation produced the Glc-Cer^−^ ion (*m/z* 810.71) and the ion at *m/z* 648.49 confirming the ceramide residue d18:1/24:0 (Figure 3b).

**Figure 4:**
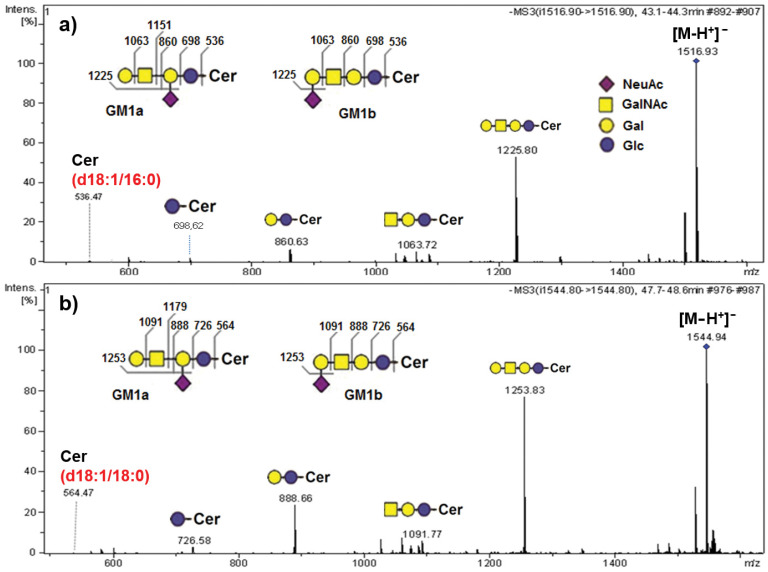
Fragmentation spectra obtained via CID MS^2^ of [M-H]^−^ ions at *m/z* 1516.93 (**a**) and *m/z* 1544.94 (**b**) confirming GM1a and GM1b isomer structures with d18:1/16:0 (**a**) and d18:1/18:0 (**b**) ceramide compositions in a ganglioside mixture from anaplastic ganglioglioma tumor tissue.

The CID MS^2^ analysis of the molecular [M-H]^-^ ion at *m/z* 1516.93 corresponding to the GM1 ganglioside with the d18:1/16:0 ceramide composition, isolated from the AGGL tissue GG mixture, revealed the possible presence of GM1a and GM1b isomers in a mixture with a d18:1/16:0 ceramide composition (Figure 4a). The MS^2^ analysis of the [M-H]^−^ ion at *m*/*z* 1544.94 revealing again a possible GM1a and GM1b isomers mixture with a d18:1/18:0 ceramide composition is shown in parallel (Figure 4b). The fragmentation spectra showed ions corresponding to fragments of GM1a and GM1b isomers: Gal-GalNAc-Gal-Glc-Cer at *m*/*z* 1225.80 (Figure 4a) and *m/z* 1253.83 (Figure 4b), GalNAc-Gal-Glc-Cer at *m/z* 1063.72 (Figure 4a) and *m/z* 1091.77 (Figure 4b), Gal-Glc-Cer at *m/z* 860.63 (Figure 4a) and at *m/z* 888.66 (Figure 4b), Glc-Cer at *m/z* 698.62 (Figure 4a) and *m/z* 726.58 (Figure 4b), and ceramide at *m/z* 536.47 (Figure 4a) and *m/z* 564.47 (Figure 4b).

**Figure 5:**
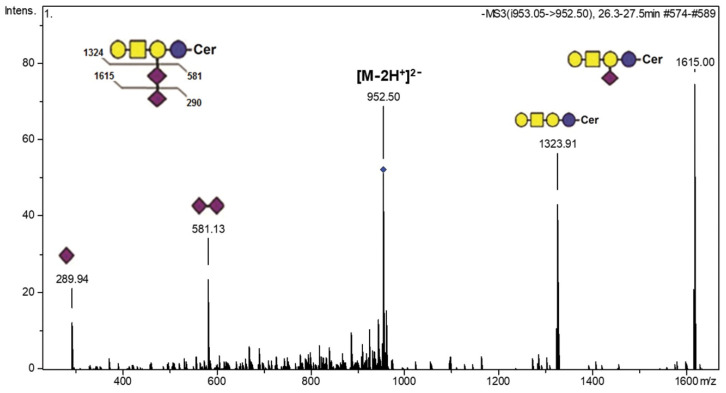
Fragmentation spectra obtained via CID MS^2^ of the [M-2H^+^]^2−^ ion at *m/z* 952.50 confirming the GD1b (d18:1/23:0) structure in the ganglioside mixture from anaplastic ganglioglioma tumor tissue.

The fragmentation spectra of the [M-2H^+^]^2−^ ion at *m/z* 952.50 from the AGGL sample GG mixture correspond to the GD1b (d18:1/23:0) ganglioside (Figure 5, Table S1). The ions detected at *m/z* 1615.00 and 1323.91 correspond to NeuAc-Gal-GalNAc-Gal-Glc-Cer^−^ and Gal-GalNAc-Gal-Glc-Cer^−^ residues, respectively. Product ions detected at *m/z* 289.94 and *m/z* 581.13 correspond to monosialo and disialo fragments (NeuAc^−^ and NeuAc-NeuAc^−^), respectively. The ion at *m/z* 581.13 confirms the presence of the GD1b isomer, while the fragment ion at *m/z* 289.94 could be produced from both isomers, GD1a and GD1b. Although the presence of the GD1a isomer cannot be completely excluded from the spectrum data, the fact that this fraction was not observed via HPLC separation corroborates the conclusion for the presence of the GD1b isomer.

### 2.5. Structural Characterization of Sphingoid Bases Obtained from AGGL Gangliosides Ceramides

**Figure 6:**
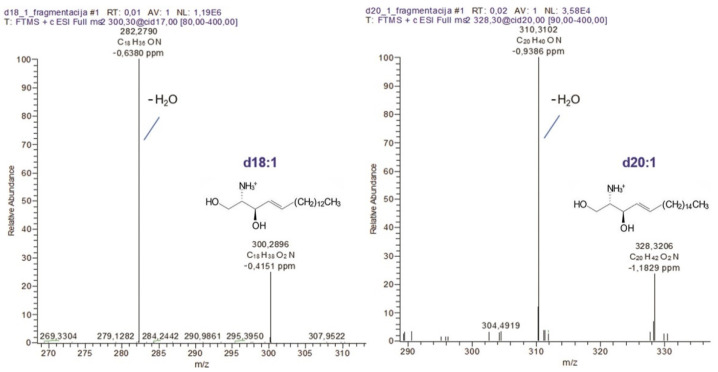
Fragmentation spectra obtained via HR MS^2^ of positively charged molecular ions [M+H^+^]^+^ at *m/z* 300.2891 and 328.3201 confirming sphingosine (d18:1) and eicosasphingosine (d20:1) structures from anaplastic ganglioglioma tumor ganglioside extract.

CID fragmentation via high-resolution (HR) mass spectrometry of sphingoid bases (obtained via hydrolytic cleavage from acidic glycosphingolipids) revealed that they fragment via single dehydration (Figure 6). Fragmentation spectra (MS^2^) of positively charged molecular ions [M+H^+^]^+^ at *m/z* 300.2896 and 328.3206 that correspond to sphingosine (d18:1) and eicosasphingosine (d20:1) are shown in Figure 6. Fragment ions at *m/z* 282.2790 and *m/z* 310.3102 correspond to dehydrated parent ions d18:1-H_2_O and d20:1-H_2_O, respectively. Mass accuracy was within the error of ±1.2 ppm. Theoretical and experimental *m/z* values of characterized sphingoid bases, their molecular formulas, and ppm accuracy values are shown in Table 2.

## 3. Discussion

Changes in ganglioside composition in different types of brain tumors are accompanied by higher expression of specific ganglioside species. “Simple” gangliosides GD2 and GD3 are considered as the glioma-associated antigens [26,27,28] and used in antitumor therapy development and clinical applications [29,30]. For example, GD3 is a potential therapeutic target for GBM treatment because of its high expression at the tumor cells and findings that both GD3 and GD3 synthase play important roles in tumorigenesis of GBM [28]. GD2 is strongly overexpressed particularly in neuroblastoma and gliomas, while its expression in normal tissues is very limited [30], which led to the development of anti-GD2 monoclonal antibodies currently in use as therapy for the pediatric population with high risk neuroblastoma [31,32]. In addition, one of the most promising novel therapeutic approaches is the chimeric antigen receptor T (CAR-T) cell therapy with CAR-T cells directed against GD2 in neuroblastoma [33] and diffuse midline gliomas, aggressive and fatal pediatric brain cancers [29]. In addition, the ganglioside profile of human gliomas has been correlated to the histomorphology and malignancy grading. In high-grade malignancy glioma, GBM, the abundance of GD2, and GD3 ganglioside species were correlated to the proliferative properties and median survival time [34]. 

According to our knowledge, ganglioside analysis of AGGL has not been performed so far. As we documented here, this rare neuronal–glial tumor type is characterized by highly altered ganglioside composition compared to peritumoral and normal brain tissues, including significant changes in both carbohydrate and ceramide backbones of ganglioside species. Although classified as a neuronal–glial type of tumor, AGGL qualitative and quantitative compositions of gangliosides showed resemblance to the compositions of high-grade malignancy gliomas, GBM [35], and gliosarcoma [36] previously characterized by our group.

The most prominent difference when comparing AGGL to PT and NB tissues was a very low total content of GGs, 4.6 and 5.7 times lower, respectively, which possibly reflects the highly invasive nature of the tumor that transformed in only six months from low malignancy ganglioglioma (grade I and Ki-67 index 3%) to anaplastic ganglioglioma (grade III and Ki-67 index 25%). Low GG content was also the feature of GBM [35] and gliosarcoma [35] specimens we had analyzed earlier. Another important hallmark of AGGL tumor tissue was a profoundly higher relative abundance of simple GG species GD3 and GM3, denoted as glioma-associated [37,38] and considered as “onco-fetal” due to their higher expression in fetal brain tissue and involvement in brain cell growth during fetal brain development [39]. Moreover, most of the AGGL ganglioside classes were represented by a larger number and relative proportion of individual species with distinct ceramide backbones than detected in PT or NB tissues, the tumor characteristic which we had previously documented in GBM [35].

The most abundant ganglioside species detected via HPTLC analysis correspond to GD3, GD2, and GM3 appearing as double bands due to the diversity in ceramide composition. This phenomenon was previously seen in different types of tumors such as neuroblastoma [40] and GBM [35]. The MS analysis of the GG mixture confirmed the variability in ceramide composition (Figure 3), showing that the most abundant GD3 class is a mixture of different GD3 ganglioside species containing diverse ceramide backbones, as previously found in GBM [35]. The most abundant sphingoid base detected in the ceramide backbones of all ganglioside classes in AGGL was sphingosine (d18:1), followed by eicosasphingosine (d20:1) (Figure 6, Table 2). Among GSLs of all mammalian tissues, only nervous-tissue gangliosides contain a significant amount of eicosasphingosine (d20:1), a proportion of which increases with aging and reaches up to 60–70% of all sphingoid bases in gangliosides, while representing only 10% in early development [41]. In AGGL, high variability was detected in the length of fatty acyl residues of the ceramide backbones of gangliosides, varying from 16 to 24 C-atoms, with the most abundant ion corresponding to GD3 with d18:1/24:0 ceramide residue containing sphingosine (d18:1) and 24:0 fatty acid, as structurally identified by MS^n^ fragmentation (Figure 3). Other ceramide backbones of AGGL gangliosides contained saturated fatty acids C16:0 (Figure 4), C22:0, and C23:0 (Figure 5), and monounsaturated C24:1 fatty acid (Figure 2 and Table S1, Supplementary Materials).

As mentioned, different ceramide species have distinct cellular functions. For example, ceramides d18:1/16:0 and d18:1/18:0 are associated with induction of apoptosis, while those with longer fatty acid chains (C22, C24) show antiapoptotic properties [16,17]. Generally, the sphingolipid ceramide has an essential role in the regulation of the mitochondrial part of the apoptosis process and signaling [42]. In mammalian cells, six different ceramide synthases (CerS1–6, also referred to as “sphinganine *N*-acyl-transferases”) catalyze de novo the synthesis of ceramides, generating ceramides with distinct fatty acid chain lengths, varying from C14 to C26 [43]. The most highly expressed CerS in the central nervous system is CerS1, which generates mainly ceramides with a fatty acid chain containing 18 C-atoms (C18-ceramide); higher abundance of fatty acids with longer and shorter chains in ceramides of tumor cells is a result of higher activity of particular CerSs, such as CerS2, which predominantly catalyze the biosynthesis of ceramides containing very long-chain fatty acids with 22 and 24 C-atoms [44]. In healthy brain tissue, CerS2 is transiently expressed in oligodendrocytes during active myelination [16]. Further on, a higher content of ceramides with long and very long fatty acid chains in skull base tumors was reported to correlate with more aggressive behavior of tumor cells [45]. On the other hand, a 70% reduction of C18-ceramide content was reported in GBM compared to normal brain tissue [46], while overexpressed CerS1 or exogenously added C18-ceramides showed inhibition of cell viability and induced apoptosis in cultured human glioma cells [47]. The mechanism by which ceramides with variable chain length influence cell behavior has not been elucidated yet, but one explanation could be interaction with signaling pathways; very long-chain fatty acids are longer than the length of the lipid monolayer and therefore protrude into the cytoplasmic layer of the membrane bilayer and influence signaling pathways via interactions with membrane proteins at the cytoplasmic part of the membrane [18]. Grösch et al. [16] hypothesized that shorter ceramides have a higher capacity of mixing with cholesterol and thus influence the lipid rafts‘ capability to activate apoptotic pathways, while longer ceramides such as C24:0 do not have this capacity, which could explain their proliferative influence. Similarly, fatty acids with an odd number of C-atoms possibly have some important roles in tumor cell survival and viability since their content in normal human brain sphingolipids is very low or detected only in trace amounts [48].

### Study Limitations

A single anaplastic ganglioglioma tissue specimen was used in this research and therefore could not be assumed as typical to AGGL tumors in general. Nevertheless, due to the very low incidence of this tumor, we believe that any data on this rare tumor type could be of interest to the scientific community, especially analyzed in comparison with valuable but often not accessible sample of peritumoral tissue.

## 4. Materials and Methods

### 4.1. Sampling and Characterization of Tumor, Peritumoral Tissue, and Normal Brain Tissue

A female patient (46 years old) was diagnosed with a brain tumor in the left hippocampal region after several years of strong headaches, occasional aphasia, and an epileptic seizure three months before the diagnosis. The tumor was surgically removed, and histopathological examination determined that it consisted of atypical glial cells, with glial fibrillary acidic protein (GFAP) and vimentin-positive cytoplasm, and atypical ganglial and ganglioidal cells showing CD34, chromogranin, and synaptophysin immunoreactivity. The proliferative index (Ki-67) was 3%, and the tumor was characterized as ganglioglioma grade I (WHO 2016) [1]. Six months after the initial surgery, the patient experienced two epileptic attacks, one type grand mal, and the second partial type, with strong headaches and visual impairment. Control magnetic resonance imaging (MRI) of the brain revealed a recurrent tumor in the left temporal apical region. The patient was subjected to a second surgery with total removal of the tumor. Samples of tumor and peritumoral tissues obtained during the surgery were stored separately in liquid nitrogen until the analysis.

Histopathological examination of the recurrent tumor revealed hemorrhagic tumorous tissue composed of atypical and mitotically active glial cells with numerous tumor blood vessels, microvascular proliferation, a small region of necrosis, and micro-calcifications. Most of the tumor cells showed GFAP immunoreactivity, and some showed synaptophysin immunoreactivity. The tumor cells showed vimentin immunoreactivity with no chromogranin, neurofilament, and isocitrate dehydrogenase 1 (IDH-1) immunoreactivity. The Ki-67 proliferative index of the recurrent tumor was up to 25%. The tumor was characterized as anaplastic ganglioglioma grade III (WHO 2016) [1]. The surgical procedures and histopathological examinations were performed in the Department of Neurosurgery and Clinical Department of Pathology “Ljudevit Jurak”, University Hospital Center “Sestre milosrdnice”, Zagreb, Croatia.

Peritumoral normal brain tissue in the anaplastic ganglioglioma patient, which could be used as a healthy control sample, was comprising the eloquent mesial temporal lobe region, making it inadequate for tissue sampling due to its role in preserving the patient’s cognitive functionality. The brain sample punch included the cortical and subcortical part of the right hemisphere temporo-occipital region from a healthy subject (78 years old) deceased in a traffic accident and was used as healthy control. This sample was obtained from the Department of Forensic Medicine, School of Medicine, University of Zagreb, Croatia, without any signs of pathological processes.

### 4.2. Gangliosides Extraction and Purification

Crude ganglioside mixtures from anaplastic ganglioglioma, peritumoral tissue, and normal brain tissue were extracted and purified according to the method of Svennerholm and Fredman [49], modified in our laboratory [50] and used in our previous studies of brain tumor gangliosides [35,36,51,52]. Briefly, weighed tissue samples were homogenized in ice-cold redistilled water (W) obtaining 10% water homogenate. The solvent system of chloroform (C), methanol (M), and water in the final volume ratio 1:2:0.75 was used for both extraction and re-extraction of lipids. Partitioning and repartitioning was performed on the pooled lipid extracts. The combined upper phases containing polar GSL were collected and evaporated to dryness prior to further purification. The alkaline hydrolysis step was avoided during purification in order to preserve alkali-labile species. Dried GG extract was purified from salts and residual denatured proteins via gel filtration on Sephadex-G25 (Sigma-Aldrich, St Louis, MO, SAD) and divided into aliquots for further GG analysis and for sphingoid base (SB) extraction. Aliquots were evaporated to dryness and stored at −80 °C until further analysis.

### 4.3. Quantitative GG Mixture Analysis

Purified GG mixtures isolated from AGGL, PT, and NB tissues were analyzed in parallel. GG content was quantitatively determined according to the spectrophotometric method of Svennerholm [53] as modified by Miettinen and Takki-Luukkainen [54]. Standard solutions of *N*-acetylneuraminic acid (sialic acid, SA) (Sigma-Aldrich, St. Louis, MO, USA, SAD) were prepared in a range of known concentrations. Absorbances of both standards and samples were determined at 580 nm (Varian Cary Bio 100, Varian Inc., Palo Alto, CA, USA, SAD). GG content in samples was expressed in microgram of GG-bound sialic acid (GG-SA) per gram of fresh tissue wet weight (w.w.).

### 4.4. High-Performance Thin-Layer Chromatography Analysis of GG Mixtures

GG mixtures were analyzed via HPTLC on silica gel plates (silica-gel 60, 10 × 10 cm, Merck, Darmstadt, Germany). Samples were prepared by dissolving evaporated extracts in C:M:W (60:30:4.5, by vol.) to contain 10 μg of GG-SA and were applied on a HPTLC plate. The plates were developed in a mobile phase containing C, M, and 0.2% aq CaCl_2_ (50:40:10, by volume). The dried plate was sprayed with resorcinol reagent and heated for 50 min at 110 °C. Visualized GG fractions were densitometrically scanned and quantified using the *ImageJ* software (NIH, Bethesda, MD, USA, SAD) as the relative proportion (%) of each GG fraction in a sample.

### 4.5. MS Characterization of Ganglioside Mixtures

Mass spectrometry screening and structural characterization of GG mixtures by tandem MS experiments were acquired on Bruker amaZon ETD ion trap system (Bruker Daltonik GmbH, Bremen, Germany) with an Apollo electrospray ionization source. The samples were prepared by dissolving evaporated GG mixtures in HPLC-grade methanol (Merck, Darmstadt, Germany) to a 0.8 µM concentration of GG-bound sialic acids and loaded by direct injection at a flow rate of 60 µL/h. The electrospray capillary voltage was set at 4600 V, the temperature of the drying gas at 210 °C, and flow rate at 5 L/min. CID excitation time was 40 ms and the amplitude was in the 0.4 to 1 V range. All spectra were acquired in negative ion mode using a scan range from *m/z* 250 to 2800. Helium was used as the collision gas. The DataAnalysis 4.0 software (Bruker Daltonik GmbH, Bremen, Germany) was used for crude analysis and extraction of the MS and MSMS data, while for GG identification using *m/z* values of GG parent and fragmentation ions we used the *GSL finder* application and database developed by our group [55].

### 4.6. Sphingoid Base Extraction and Purification

Sphingoid bases (SBs) were extracted from GG extracts isolated from AGGL sample, PT, and NB tissues. Purified and dried GG extracts were subjected to acid hydrolysis using aqueous methanolic HCl [56] to release SBs from acidic GSLs (gangliosides and sulfated GSLs) via hydrolytic cleavage of glycosidic linkages and of amide bonds linking SBs and fatty acyl residues in GG ceramides. The starting equivalent mass of each tissue homogenate subjected to acid hydrolysis as purified GG extract was 60 mg. Isolated sphingoid bases were structurally characterized using high-resolution mass spectrometry (HR-MS) fragmentation.

### 4.7. HR-MS Characterization of Sphingoid Bases

High-resolution mass spectrometry experiments were performed on an LTQ-Orbitrap Discovery (Thermo Fisher Scientific, Waltham, MA, USA). Samples were dissolved in methanol and loaded (100 μL injection volume) via direct injection to MS in a 1 min run. The electrospray capillary voltage was set at 5.1 kV, the capillary temperature at 275 °C, the instrument resolution was 100,000 at 400 *m/z*, and mass accuracy was within the error of ±5 ppm. Tandem MS experiments were performed using CID. Mass range was set from 80 to 400 *m/z* and the isolation width was 1 *m/z* with a normalized collision energy of 20 V and activation time of 30 ms. Helium was used as the collision gas.

## 5. Conclusions

According to our knowledge, this is the first characterization of gangliosides in anaplastic ganglioglioma, a neuronal–glial type of tumor. AGGL ganglioside composition was found to be highly altered as compared to peritumoral (PT) and normal brain (NB) tissues, including changes in both carbohydrate and ceramide residues of ganglioside species. The evidenced qualitative and quantitative specificities of AGGL ganglioside composition were similar to the characteristics of GBM [35] and gliosarcoma [36], high-grade malignancy gliomas, analyzed earlier by our group. In all three tumor entities, the simple so called “onco-fetal”, ganglioside species were the most abundant species. Additionally, in all three entities, larger variability of ganglioside ceramide structures with considerable relative abundance was observed as compared to both peritumoral and normal brain tissues in which ganglioside ceramide residues d18:1/18:0 and d20:1/18:0 were highly dominant relative to the other ones composing a particular ganglioside class. We believe that in further research of ganglioside-related metabolism in cancer cells we should pay at least as much attention to their ceramide portions’ structural variability as we do to the antigenic carbohydrate part, the importance of which is so far better documented.

The additional contribution of this study is the characterization of the ganglioside composition in valuable and rarely accessible peritumoral tissue since the important biochemical changes that occur within this area are considered responsible for tumor cell infiltration into the surrounding healthy brain tissue promoting tumor recurrence and possibly for tumor resistance to chemotherapeutic treatment [23,57].

We also believe that our experimental approach, combining complementary methods, employed in this and our previous work is useful in demonstrating the realistic GG expression profile of investigated tumors as well as in identifying the major GG species acting as cell-surface antigens of specific tumor specimens.

## Figures and Tables

**Table 1:** Relative proportions of HPTLC-separated GG fractions from anaplastic ganglioglioma (AGGL), peritumoral tissue (PT), and normal brain tissue (NB).

Ganglioside Fractions (%) Migrating as:	AGGL	PT	NB
GM3 ^a^	6.2	1.3	3.1
GM2	n.d.	4.7	2.2
X2	1.5	n.d.	n.d.
GM1	1.4	23.4	15.7
GD3 ^a^	52.4	4.0	9.7
GD1a	n.d.	22.3	17.3
X1	6.1	n.d.	n.d.
GD2 ^a^	12.1	2.1	6.4
GD1b	4.0	19.4	20.0
*O*-Ac-GT1b	n.d.	1.5	2.8
GT1b	11.3	19.2	20.7
GQ1b	4.7	2.1	1.8
GP1	0.4	n.d.	n.d.

^a^ both upper and lower bands; X2—possibly 3′-isoLM1; X1—possibly GD1α. Total ganglioside content (μg GG-SA/g tissue w.w.): 96.4 in AGGL, 447.0 in PT, and 545.2 in NB.

**Table 2:** Theoretical and experimental *m/z* values and the corresponding molecular formulas of positively charged precursor and dehydrated fragment ions of sphingosine (d18:1) and eicosasphingosine (d20:1) isolated from the AGGL acidic GSL mixture and characterized by HR-MS CID tandem MS. Mass accuracy is expressed in ppm.

Sphingoid Base Type	*m/z*			Molecular Formula	*m/z*			Molecular Formula
	Theor.	Experim.	ppm		Theor.	Experim.	ppm	
	[M+H^+^]^+^				[M+H^+^-H_2_O]^+^			
d18:1	300.2897	300.2896	−0.4151	C_18_H_38_O_2_N	282.2786	282.2790	−0.6380	C_18_H_36_ON
d20:1	328.3210	328.3206	−1.1829	C_20_H_42_O_2_N	310.3104	310.3102	−0.9386	C_20_H_40_ON

## Supplementary Materials

The following are available online at https://www.mdpi.com/article/10.3390/ijms22168844/s1.

## Abbreviations

Gangliosides and the precursor glycosphingolipids are abbreviated according to the system of Svennerholm [58,59,60] and the recommendations of the IUPAC-IUB Commission on Biochemical Nomenclature [61] as follows:

**LacCer**	Galβ4Glcβ1Cer;
**GA2**	Gg_3_Cer, GalNAcβ4Galβ4Glcβ1Cer;
**GA1**	Gg_4_Cer, Galβ3GalNAcβ4Galβ4Glcβ1Cer;
**Lc_3_**	GlcNAcβ3Galβ4Glcβ1Cer;
**Lc_4_**	Galβ3GlcNAcβ3Galβ4Glcβ1Cer;
**nLc_4_**	Galβ4GlcNAcβ3Galβ4Glcβ1Cer;
**nLc_6_**	Galβ4GlcNAcβ3Galβ4GlcNAcβ3Galβ4Glcβ1Cer;
**Gb_3_**	Galα4Galβ4Glcβ1Cer;
**Gb_4_**	GalNAcβ3Galα4Galβ4Glcβ1Cer;
**iGb_3_**	Galα3Galβ4Glcβ1Cer;
**iGb_4_**	GalNAcβ3Galα3Galβ4Glcβ1Cer;
**GM3**	II^3^-α-Neu5Ac-LacCer;
**GD3**	II^3^-α-(Neu5Ac)_2_-LacCer;
**GT3**	II^3^-α-(Neu5Ac)_3_-LacCer;
**GM2**	II^3^-α-Neu5Ac-Gg_3_Cer;
**GD2**	II^3^-α-(Neu5Ac)_2_-Gg_3_Cer;
**GM1a** or **GM1**	II^3^-α-Neu5Ac-Gg_4_Cer;
**GM1b**	IV^3^-α-Neu5Ac-Gg_4_Cer;
**LM1**	IV^3^-α-Neu5Ac- Lc_4_Cer;
**nLM1**	IV^3^-α-Neu5Ac- nLc_4_Cer;
**GD1a**	IV^3^-α-Neu5Ac,II^3^-α-Neu5Ac-Gg_4_Cer;
**GD1b**	II^3^-α-(Neu5Ac)_2_-Gg_4_Cer;
**LD1**	IV^3^-α-Neu5Ac,II^6^-α-Neu5Ac-Lc_4_Cer;
**nLD1**	IV^3^-α-Neu5Ac,II^6^-α-Neu5Ac-nLc_4_Cer;
**GT1b**	V^3^-α-Neu5Ac,II^3^-α-(Neu5Ac)_2_-Gg_4_Cer;
**GQ1b**	IV^3^-α-(Neu5Ac)_2_,II^3^-α-(Neu5Ac)_2_-Gg_4_Cer.
