# Supplementary material for: Ganglioside Composition Distinguishes Anaplastic Ganglioglioma Tumor Tissue from Peritumoral Brain Tissue: Complementary Mass Spectrometry and Thin-Layer Chromatography Evidence
Source: Int J Mol Sci. 2021 Aug 17;22(16):8844. doi: 10.3390/ijms22168844 (PMC8396361; doi:10.3390/ijms22168844)
Supplement: Supplementary file 1 [file ijms-22-08844-s001.zip › Supplement_Figure S1. MS1 screening (AGGL, PT, NB)_fin.pdf]

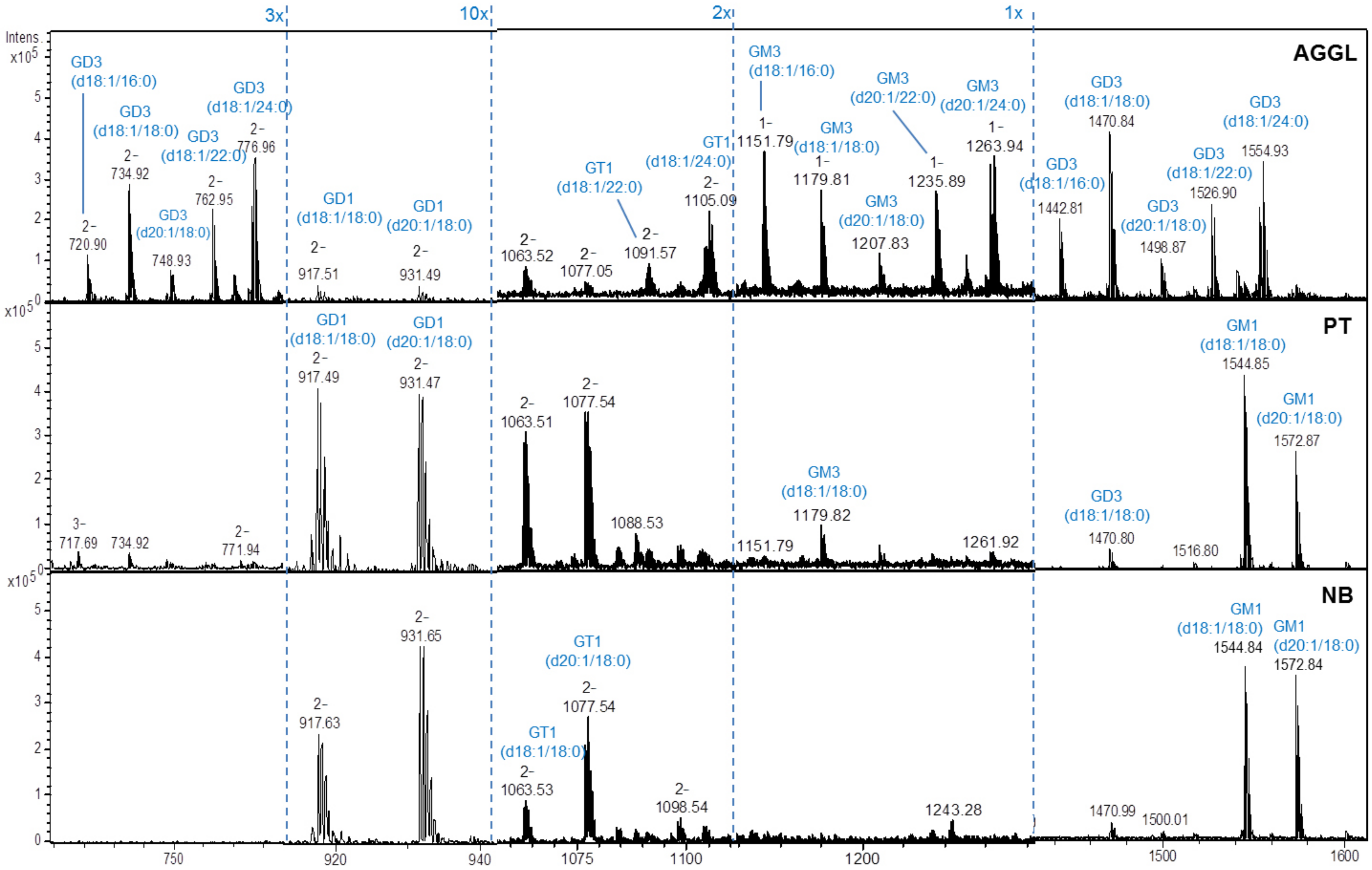

Figure S1. Negative ion mode MS1 screening of the native ganglioside mixture from anaplastic ganglioglioma (AGGL), peritumoral tissue (PT) and normal brain tissue (NB). Corresponding molecular species and ion abundances are presented in Tables S1, S2 and S3 (Supplementary Data).
