# Supplementary material for: Ganglioside Composition Distinguishes Anaplastic Ganglioglioma Tumor Tissue from Peritumoral Brain Tissue: Complementary Mass Spectrometry and Thin-Layer Chromatography Evidence
Source: Int J Mol Sci. 2021 Aug 17;22(16):8844. doi: 10.3390/ijms22168844 (PMC8396361; doi:10.3390/ijms22168844)
Supplement: Supplementary file 1 [file ijms-22-08844-s001.zip › Supplement_Table S1. AGGL (MS1 ion list)_fin.pdf]

Table S1. Negatively charged molecular ions corresponding to ganglioside species detected by MS analysis of native ganglioside mixture isolated from anaplastic ganglioglioma (AGGL) tumor tissue.

m/z		Gangliosides Molecular species	Ion intensity	
$[M\text{-}2H^+]^{2-}$	$[M\text{-}H^+]^-$	Anaplastic Ganglioglioma (AGGL)	Sum	% of max
	1151.79	GM3 (d18:1/16:0)	62987	29.11
	1179.81	GM3 (d18:1/18:0)	45474	21.02
	1207.83	GM3 (d20:1/18:0) and/or (d18:1/20:0)	19929	9.21
	1233.83	GM3 (d18:1/22:1)	14883	6.88
	1235.81	GM3 (d18:1/22:0)	40281	18.62
	1249.87	GM3 (d18:1/23:0)	16849	7.79
	1261.88	GM3 (d18:1/24:1)	46906	21.68
	1263.94	GM3 (d18:1/24:0)	46396	21.44
	1354.79	GM2 (d18:1/16:0)	7159	3.31
	1382.82	GM2 (d18:1/18:0)	7971	3.68
720.90	1442.81	GD3 (d18:1/16:0)	98569	45.55
727.84	1456.68	GD3 (d18:1/17:0)	6110	2.82
733.90	1468.82	GD3 (d18:1/18:1)	10733	4.96
734.92	1470.84	GD3 (d18:1/18:0)	111963	51.74
741.88	1484.81	*O*-Ac-GD3 (d18:1/16:0)	5149	2.38
748.93	1498.86	GD3 (d20:1/18:0) and/or (d18:1/20:0)	54121	25.01
761.88	1524.77	GD3 (d18:1/22:1)	9121	4.22
762.95	1526.90	GD3 (d18:1/22:0)	144088	66.59
769.88	1540.87	GD3 (d18:1/23:0) and/or *O*-Ac-GD3 (d20:1/18:0)	45265	20.92
775.95	1552.82	GD3 (d18:1/24:1)	142676	65.94
776.91	1554.93	GD3 (d18:1/24:0)	216383	100.00
757.87	1516.84	GM1 (d18:1/16:0)	9307	4.30
771.93	1544.86	GM1 (d18:1/18:0)	12390	5.73
	1572.90	GM1 (d20:1/18:0) and/or (d18:1/20:0)	10309	4.76
	1626.97	GM1 (d18:1/24:1)	9546	4.41
822.69	1645.89	GD2 (d18:1/16:0)	15666	7.24
836.45	1673.91	GD2 (d18:1/18:0)	54837	25.34
850.50	1701.94	GD2 (d20:1/18:0) and/or (d18:1/20:0)	14010	6.47
864.48	1729.97	GD2 (d18:1/22:0)	25158	11.63
877.61	1756.23	GD2 (d18:1/24:1)	27520	12.72
878.62	1758.25	GD2 (d18:1/24:0)	30589	14.14
917.51	1835.96	GD1 (d18:1/18:0)	57375	26.52
931.49	1863.99	GD1 (d20:1/18:0) and/or (d18:1/20:0)	46369	21.43
945.49	1892.03	GD1 (d18:1/22:0)	29181	13.49
952.50	1906.01	GD1 (d18:1/23:0)	18047	8.34
958.50	1918.04	GD1 (d18:1/24:1)	30484	14.09
959.50	1920.00	GD1 (d18:1/24:0)	42879	19.82
976.50	1954.01	Fuc-GD1 (d18:1/16:0)	11000	5.08
1033.03	2067.07	HexNAc-GD1 (d20:1/18:0) and/or (d18:1/20:0)	12187	5.63
1049.00	2099.01	GT1 (d18:1/16:0)	11251	5.20
1063.52	2127.06	GT1 (d18:1/18:0)	22154	10.24
1077.05	2155.09	GT1 (d20:1/18:0) and/or (d18:1/20:0)	17381	8.03
1091.57	2183.12	GT1 (d18:1/22:0)	17655	8.16
1098.03	2197.13	GT1 (d18:1/23:0)	11252	5.20
1104.03	2209.08	GT1 (d18:1/24:1)	30179	13.95
1105.09	2211.08	GT1 (d18:1/24:0)	43062	19.90
