# Supplementary material for: Ganglioside Composition Distinguishes Anaplastic Ganglioglioma Tumor Tissue from Peritumoral Brain Tissue: Complementary Mass Spectrometry and Thin-Layer Chromatography Evidence
Source: Int J Mol Sci. 2021 Aug 17;22(16):8844. doi: 10.3390/ijms22168844 (PMC8396361; doi:10.3390/ijms22168844)
Supplement: Supplementary file 1 [file ijms-22-08844-s001.zip › Supplement_Table S2. PT (MS1 ion list)_fin.pdf]

Table S2. Negatively charged molecular ions corresponding to ganglioside species detected by MS analysis of native ganglioside mixture isolated from peritumoral tissue (PT).

m/z			Molecular species of GG	Ion intensity	
$[M-3H^+]^{3-}$	$[M-2H^+]^{2-}$	$[M-H^+]^-$	Peritumoral Tissue (PT)	Sum	% of max
		1151.79	GM3 (d18:1/16:0)	8130	1.38
		1179.82	GM3 (d18:1/18:0)	31843	5.41
		1207.80	GM3 (d20:1/18:0) and/or (d18:1/20:0)	13720	2.33
		1233.83	GM3 (d18:1/22:1)	9090	1.54
		1261.92	GM3 (d18:1/24:1)	11310	1.92
		1380.77	GM2 (d18:1/18:1)	7132	1.21
		1382.82	GM2 (d18:1/18:0)	44960	7.64
		1410.85	GM2 (d20:1/18:0) and/or (d18:1/20:0)	17844	3.03
		1464.95	GD3 (d18:1/16:0)^{Na}	3543	0.60
	734.92	1470.80	GD3 (d18:1/18:0)	46101	7.83
	748.88	1498.86	GD3 (d20:1/18:0) and/or (d18:1/20:0)	4794	0.81
	761.88	1524.77	GD3 (d18:1/22:1)	20014	3.40
	762.90	1526.81	GD3 (d18:1/22:0)	16062	2.73
	757.87	1516.80	GM1(d18:1/16:0)	8870	1.51
	771.94	1544.85	GM1(d18:1/18:0)	266812	45.33
		1572.87	GM1 (d20:1/18:0) and/or (d18:1/20:0)	157918	26.83
		1600.93	GM1 (d18:1/22:0)	10241	1.74
	804.77	1610.54	GM1 (d18:1/24:0)-H_2O	21375	3.63
		1626.97	GM1 (d18:1/24:1)	4699	0.80
		1629.00	GM1 (d18:1/24:0)	3923	0.67
	836.45	1673.91	GD2 (d18:1/18:0)	106295	18.06
	850.47	1701.94	GD2 (d20:1/18:0) and/or (d18:1/20:0)	22956	3.90
	917.49	1835.96	GD1 (d18:1/18:0)	588627	100.00
	931.47	1863.99	GD1 (d20:1/18:0) and/or (d18:1/20:0)	568515	96.58
	938.47	1877.95	*O*-Ac-GD1 (d18:1/18:0)	26977	4.58
	945.49	1892.03	GD1 (d18:1/22:0)	55251	9.39
	949.46	1899.94	GD1 (d18:1/24:1)-H_2O	35150	5.97
	952.44	1906.04	*O*-Ac-GD1 (d20:1/18:0)	36533	6.21
	958.50	1918.04	GD1(d18:1/24:1)	22169	3.77
	959.50	1920.00	GD1 (d18:1/24:0)	23151	3.93
	966.43	1933.87	*O*-Ac-GD1 (d18:1/22:0)	35192	5.98
	980.93	1961.87	*O*-Ac-GD1 (d18:1/24:0)	47368	8.05
	999.45	1999.91	Hex-GD1 (d18:0/18:0)	10205	1.73
	1018.97	2039.04	HexNAc-GD1 (d18:1/18:0)	15156	2.57
	1033.03	2067.07	HexNAc-GD1 (d20:1/18:0) and/or (d18:1/20:0)	12487	2.12
	1039.47	2080.09	Hex-GD1 (d18:1/24:1)	12776	2.17
	1053.51	2108.02	Hex-GD1 (d20:1/24:1)	11491	1.95
708.35	1063.51	2127.06	GT1 (d18:1/18:0)	157625	26.78
717.69	1077.54	2155.09	GT1 (d20:1/18:0) and/or (d18:1/20:0)	165248	28.07
	1084.03	2169.07	*O*-Ac-GT1 (d18:1/18:0)	*20515*	3.49

726.97	1091.00	2183.12	GT1 (d18:1/22:0)	20852	3.54
731.64	1098.03	2197.13	*O*-Ac-GT1 (d20:1/18:0) and/or (d18:1/20:0)	26720	4.54
	1103.04	2207.07	GT1 (d18:2/24:1)	12564	2.13
	1112.00	2225.01	*O*-Ac-GT1 (d18:1/22:0)	10196	1.73
	1125.98	2252.91	Fuc-GT1 (d18:1/18:0)-H_2O	11782	2.00
	1135.93	2272.86	Fuc-GT1 (d18:1/18:0)	12368	2.10
