# Supplementary material for: Ganglioside Composition Distinguishes Anaplastic Ganglioglioma Tumor Tissue from Peritumoral Brain Tissue: Complementary Mass Spectrometry and Thin-Layer Chromatography Evidence
Source: Int J Mol Sci. 2021 Aug 17;22(16):8844. doi: 10.3390/ijms22168844 (PMC8396361; doi:10.3390/ijms22168844)
Supplement: Supplementary file 1 [file ijms-22-08844-s001.zip › Supplement_Table S3. NB (MS1 ion list)_fin.pdf]

Table S3. Negatively charged molecular ions corresponding to ganglioside species detected by MS analysis of native ganglioside mixture isolated from normal brain (NB) tissue.

m/z			Gangliosides Molecular species	Ion intensity	
$[M-3H^+]^{3-}$	$[M-2H^+]^{2-}$	$[M-H^+]^-$	Normal Brain (NB)	Sum	% of max
		1179.76	GM3 (d18:1/18:0)	85175	6.18
		1207.78	GM3 (d20:1/18:0) and/or (d18:1/20:0)	48207	3.50
		1235.80	GM3 (d18:1/22:0)	21817	1.58
		1261.88	GM3 (d18:1/24:1)	21442	1.56
		1382.82	GM2 (d18:1/18:0)	84052	6.10
		1410.85	GM2 (d20:1/18:0) and/or (d18:1/20:0)	74681	5.42
	734.86	1470.99	GD3 (d18:1/18:0)	207758	15.08
	748.88	1498.86	GD3 (d20:1/18:0) and/or (d18:1/20:0)	96730	7.02
	755.92	1512.84	*O*-Ac-GD3 (d18:1/18:0)	19577	1.42
	762.94	1526.89	GD3 (d18:1/22:0)	32648	2.37
	776.92	1554.85	GD3 (d18:1/24:0)	20364	1.48
	757.87	1516.84	GM1 (d18:1/16:0)	14890	1.08
		1542.92	GM1 (d18:1/18:1)	22947	1.67
	771.93	1544.84	GM1 (d18:1/18:0)	273674	19.87
		1572.84	GM1 (d20:1/18:0) and/or (d18:1/20:0)	422754	30.69
		1600.93	GM1 (d18:1/22:0)	35281	2.56
	836.45	1673.91	GD2 (d18:1/18:0)	153462	11.14
	850.47	1701.94	GD2 (d20:1/18:0) and/or (d18:1/20:0)	188951	13.72
	917.63	1835.96	GD1 (d18:1/18:0)	685055	49.74
	924.47	1849.94	*O*-Ac-GD1 (d18:1/16:0)	32134	2.33
	931.65	1863.99	GD1 (d20:1/18:0) and/or (d18:1/20:0)	1377407	100.00
	938.47	1877.95	*O*-Ac-GD1 (d18:1/18:0)	39710	2.88
	945.49	1892.03	GD1 (d18:1/22:0)	131090	9.52
	949.46	1899.94	GD1 (d18:1/24:1)-H_2O	42470	3.08
		1901.94	GD1 (d18:1/24:0)-H_2O	9664	0.70
	952.44	1906.04	*O*-Ac-GD1 (d20:1/18:0) and/or (d18:1/20:0)	69850	5.07
	958.50	1918.04	GD1 (d18:1/24:1)	65526	4.76
	959.50	1920.00	GD1 (d18:1/24:0)	43911	3.19
	960.48	1921.96	GD1 (d18:0/24:0)	43337	3.15
	965.55	1932.18	GD1 (d20:1/24:1)	37256	2.70
	972.51	1946.00	*O*-Ac-GD1 (d18:1/22:1)	23557	1.71
	1019.02	2039.04	HexNAc-GD1 (d18:1/18:0)	15718	1.14
	1033.03	2067.07	HexNAc-GD1 (d20:1/18:0) and/or (d18:1/20:0)	17322	1.26
	1114.00	2229.01	Hex-HexNAc-GD1 (d20:1/18:0)	41820	3.04
708.35	1063.53	2127.06	GT1 (d18:1/18:0)	154444	11.21
717.66	1077.54	2155.09	GT1 (d20:1/18:0) and/or (d18:1/20:0)	371410	26.96
	1081.98	2165.00	GT1 (d18:1/22:0)-H_2O	37383	2.71
	1084.03	2169.07	*O*-Ac-GT1 (d18:1/18:0)	23802	1.73
726.97	1091.00	2183.12	GT1 (d18:1/22:0)	74153	5.38
	1096.01	2193.03	GT1 (d18:1/24:0)-H_2O	34306	2.49
731.64	1098.54	2197.13	*O*-Ac-GT1 (d20:1/18:0)	54944	3.99
	1103.04	2207.07	GT1 (d18:1/24:2)	86272	6.26
	1104.03	2209.08	GT1 (d18:1/24:1)	48870	3.55
	1108.97	2218.97	GT1 (d20:1/24:1)-H_2O	18890	1.37
